# Conserved carotenoid pigmentation in reproductive organs of Charophyceae

**DOI:** 10.1098/rstb.2023.0372

**Published:** 2024-09-30

**Authors:** Tim P. Rieseberg, Anja Holzhausen, Maaike J. Bierenbroodspot, Wanchen Zhang, Ilka N. Abreu, Jan de Vries

**Affiliations:** ^1^ Department of Applied Bioinformatics, Institute of Microbiology and Genetics, Goldschmidtstr. 1, University of Goettingen, Goettingen 37077, Germany; ^2^ Department of Crop Physiology, Martin Luther University Halle-Wittenberg, Institute of Agricultural and Nutritional Sciences, Betty Heimann-Str. 5, Halle (Saale) 06120, Germany; ^3^ Department of Plant Biochemistry, Albrecht Haller Institute of Plant Science, Justus-von-Liebig-Weg, University of Goettingen, Goettingen 37077, Germany; ^4^ Goettingen Center for Molecular Biosciences (GZMB), Goettingen Metabolomics and Lipidomics Laboratory, Justus-von-Liebig Weg 11, University of Goettingen, Goettingen 37077, Germany; ^5^ Department of Applied Bioinformatics, Goettingen Center for Molecular Biosciences (GZMB), Goldschmidtstr. 1, University of Goettingen, Goettingen 37077, Germany; ^6^ Department of Applied Bioinformatics, Campus Institute Data Science, University of Goettingen, Goettingen 37077, Germany

**Keywords:** streptophyte algae, Charophyceae, carotenoids, apocarotenoids, sexual reproductive structures

## Abstract

Sexual reproduction in Charophyceae abounds in complex traits. Their gametangia develop as intricate structures, with oogonia spirally surrounded by envelope cells and richly pigmented antheridia. The red—probably protectant—pigmentation of antheridia is conserved across Charophyceae. *Chara tomentosa* is, however, unique in exhibiting this pigmentation and also in vegetative tissue. Here, we investigated the two sympatric species, *C. tomentosa* and *Chara baltica*, and compared their molecular chassis for pigmentation. Using reversed phase C_30_ high performance liquid chromatography (RP-C_30_-HPLC), we uncover that the major pigments are β-carotene, δ-carotene and γ-carotene; using headspace solid-phase microextraction coupled to gas chromatography equipped with a mass spectrometer (HS-SPME-GC-MS), we pinpoint that the unusually large carotenoid pool in *C. tomentosa* gives rise to diverse volatile apocarotenoids, including abundant 6-methyl-5-hepten-2-one. Based on transcriptome analyses, we uncover signatures of the unique biology of Charophycaee and genes for pigment production, including monocyclized carotenoids. The rich carotenoid pool probably serves as a substrate for diverse carotenoid-derived metabolites, signified not only by (i) the volatile apocarotenoids we detected but (ii) the high expression of a gene coding for a cytochrome P450 enzyme related to land plant proteins involved in the biosynthesis of carotenoid-derived hormones. Overall, our data shed light on a key protection strategy of sexual reproduction in the widespread group of macroalgae. The genetic underpinnings of this are shared across hundreds of millions of years of plant and algal evolution.

This article is part of the theme issue ‘The evolution of plant metabolism’.

## 1. Introduction

Charophyceae are a class of streptophyte macroalgae with remarkable complex body plans and biochemistry studied since the eighteenth century (for a review of *Chara* as a model for plant biology, see Kurtović *et al*. [1]). Indeed, they were long assumed to be a sister to land plants until phylogenomic studies proved that the species-rich Zygnematophyceae fill that position [2–6]; Charophyceae, Coleochaetophyceae, Zygnematophyceae and land plants form together the monophylum of Phragmoplastophyta. The remarkable features of Charophyceae include survival in brackish habitats, a trait only found in Charophyceae and land plants within the Streptophyta [7,8]. Akin to land plants, Charophyceae have intricate sexual reproduction mechanisms including complex gametangia, monoecious as well as dioecious species. Reduction of processes in sexual reproduction, including the loss of sperm motility, in Zygnematophyceae is owing to conjugation as a mode of reproduction [9]. It is thus fair to assume that molecular programmes for the development of complex reproductive structures in phragmoplastophytes share some common ancestry.

Another important feature of streptophyte evolution is that it brought forth a highly specialized metabolism [10,11]. Carotenoids, C30 to C50 polyenes, occur in any photosynthetic organism [12,13] and even in some non-photosynthetic organisms like aphids [14]. The central C5 isoprenoid building blocks of these polyenes (isopentenyl diphosphate and dimethylallyl diphosphate) are derived from the methylerythritol phosphate pathway—the source of many terpenoids including, e.g. chlorophylls, plastoquinones and aforementioned carotenoids (for a review, see Rodriguez-Concepcion *et al.* [15]). Carotenoid biosynthesis is committed by the synthesis of phytoene through the action of phytoene synthase [16–19]. Conversion by desaturases like phytoene desaturase [20] and the action of several isomerases yield the central compound in carotenogenesis, all-*trans*-lycopene [21], from which through cyclization by lycopene cyclases (LCYs) [22–25] the textbook carotenoids are produced [12]. Canonical carotenoids like β-carotene are highly conserved in Viridiplantae [12,26]. At the same time carotenoids are one of the most diverse classes of species- and lineage-specific specialized metabolites with over 1000 members currently known [27], often serving similar functions (for an elaboration on the evolutionary implications, see Dadras *et al*. [28]). These highly conserved functions include their central role as antioxidants and as quenchers of reactive oxygen species (ROS) producing triplet-state chlorophyll in the photosystem II (PSII) core complex (for a review, see Telfer [29]). The hydroxylation and epoxidation products of carotenes—the xanthophyll pigments—are used in nearly every known light-harvesting complex (LHC) since they enlarge the spectrum of absorbed light compared to chlorophylls alone and participate in non-photochemical quenching by heat dissipation of light energy [30].

In streptophyte algae, studies of this crucial specialized metabolism are rather rare, but still, some remarkable lineage and species-specific occurrences and mechanisms are known [31,32]. In albedo-facing *Cosmarium* species (Zygnematophyceae), a truncated xanthophyll cycle was detected not integrating violaxanthin and thus named the antheraxanthin-zeaxanthin cycle [32]. Most Chloroplastida use 9-*cis*-neoxanthin in their LHCs [33]. Surprisingly, in *Mesostigma viride* solely, all-*trans*-neoxanthin is present and thus probably slightly altered LHCs [31]. Additionally, these algae contain the rather rare siphonaxanthin and fatty acid esters of it, as well as γ-carotene [31]. Monocyclized carotenoids like γ-carotene have important functions in photosynthetic as well as non-photosynthetic organisms including green bacteria [34] and UV and psychrotolerant yeasts [35]. Especially in the latter, high levels of γ-carotene were correlated with UV tolerance [35]. Charophyceae are well known for their red antheridia, and previous studies indicated that this coloration originates from monocyclized γ-carotene [36,37]. Monocyclized carotenoids rather rarely accumulate in the green lineage despite being a central intermediate in the biosynthesis of double-cyclized α-carotene and β-carotene (for a review, see Rodriguez-Concepcion *et al.* and Sathasivam *et al*. [15,38]).

We investigated the global differential gene expression patterns, chlorophylls, carotenoids and apocarotenoids of the two brackish Charophyceaen species, *Chara tomentosa* L. and *Chara baltica* (Hartman) Bruz. Using an optimized analytical method recently published [39], we found that three carotenoids, β-carotene, δ-carotene and γ-carotene, mainly contribute to antheridia colour. Second, the characteristic reddish colour change of *C. tomentosa* vegetative tissue (VT) during development/seasonal changes [40] originates from the same carotenoids. Additionally, we detect volatile apocarotenoids in Charophyceae. Our data shed light on the evolution of a complex metabolite-driven phenotype based on a conserved chassis of compounds and enzymes.

## 2. Results and discussion

### (a) Global gene expression profiles recover species and ecophysiological differences

**Figure 1.**
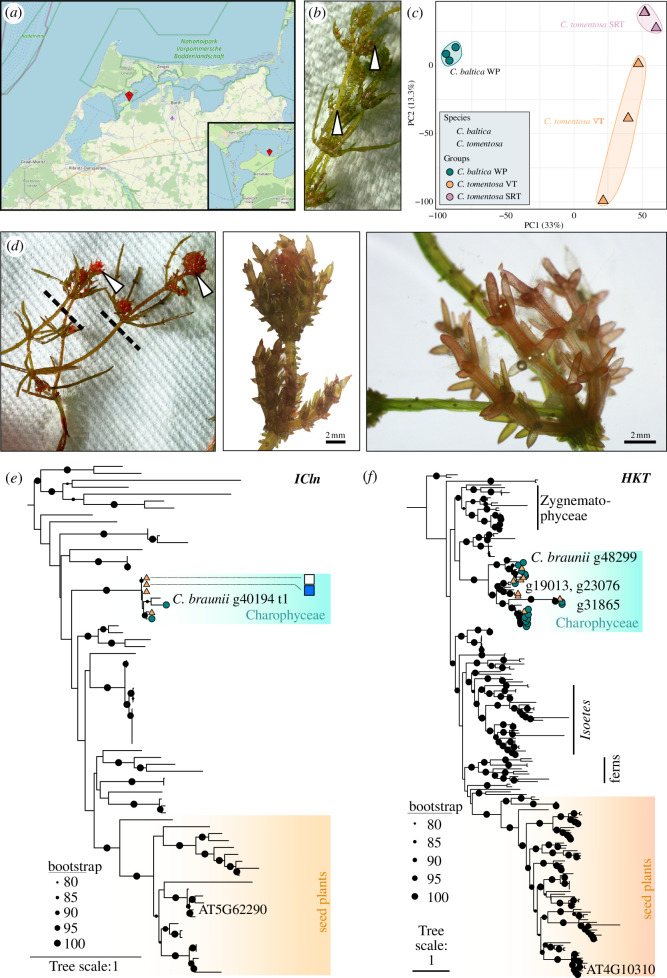
Investigation of two sympatric *Chara* species. (*a*) Location of sampling near Michaelsdorf in the brackish ‘Bodstetter Bodden’ of the Darss Zingster Bodden chain. Map from www.openstreetmap.org. (*d*) Male *C. tomentosa*, dashed line indicates cutting to obtain the two respective sample groups for this organism. On the right, two pictures show a zoom-in (of a different individual) on the morphological details of the whorls; note the spotted pigmentation (the two pictures were reproduced with permission from Holzhausen [42]). (*b*) *Chara baltica*. In (*b*) and (*d*)*,* white arrows indicate antheridia, in male *C. tomentosa* only in top parts, and in *C. baltica* distributed over the branches of the whole plant (WP). (*c*) Principal component analysis (PCA) of global differential gene expression of the three sample groups indicated by colour and shape as displayed on the right side. (*e*,*f*) Maximum-likelihood phylogenies of genes salient to salt tolerance. Black circles are scaled and represent the ultrafast bootstrap (UFBoot) support (see legend). Teal hues label highlight sequences from Charophyceae; *C. baltica* sequences are labelled with a dark turquoise circle, and *C. tomentosa* sequences are labelled with an orange triangle. Blue boxes indicate significant downregulation, and purple boxes indicate significant upregulation of a minimum of twofold in the comparison *C. tomentosa* sexual reproductive tissue (SRT) versus *C. tomentosa* VT.

The brackish waters that emerge from the influx of Baltic sea water into freshwater reservoirs are well known for their richness in Charophyceae [41]. During late spring/early summer, we sampled *C. baltica* and *C. tomentosa* from Michaelsdorf (54.371306° N, 12.569778° E), a lagoon in the Bodstedter Bodden of the Darss-Zingst Bodden chain (brackish; figure 1*a*). These species were chosen for several reasons: first, they occur sympatrically; second, *C. tomentosa* appears in brackish and freshwater habitats, while *C. baltica* is only known from brackish habitats; third, both species have the same cortication type (diplostichous) but differ in domesticity—*C. baltica* is monoecious and *C. tomentosa* is dioecious; and lastly, and salient to the specialized metabolic focus of this study, *C. tomentosa* has bodies of an astonishingly red pigmentation, suggesting high levels of carotenoids.

First, we looked at the global differential gene expression profiles of the following three sample groups: (i) *C. tomentosa* VT, (ii) antheridia-rich top parts referred to as sexual reproductive tissue (SRT; figure 1*b*), and (iii) *C. baltica* whole plants (WP; figure 1*c*). We extracted RNA from these tissues in biological triplicates and performed paired-end RNA sequencing of 150 bp length using the Illumina NovaSeq 6000 platform. We sequenced 215 491 672 reads for *C. tomentosa* VT, 218 432 320 of *C. tomentosa* apices and 191 118 580 for *C. baltica*. The reads were de novo assembled using the Trinity pipeline [43] (v. 2) into 9 89 447 and 1 368 348 contigs for *C. tomentosa* and *C. baltica*. Filtering for apt expression levels (≥1 transcripts per million (TPM)), we retained a reference of 8 57 835 contigs for *C. tomentosa*; we converted these data into predicted proteins, and after decontamination, we retained 9578 predicted proteins for *C. tomentosa* (23 099 for *C. baltica*). We mapped the reads onto our new reference assemblies using Kallisto v. 0.50.1 [44] and calculated gene expression levels using Trinity [43]; statistics were based on DESeq2 [45].

To understand the gross gene expression profiles, we conducted principal component analysis (PCA). The strongest separation was found between the two species along PC1, which described 33% of the variance in the data (see figure 1*d*). Although samples of *C. tomentosa* VT showed the overall broadest distribution along PC2—which, however, just described 13.3% of the variance—they still clearly separated from SRT-containing samples along this component. We next turned to the specific gene expression patterns therein.

Several specific gene expression patterns correlated with the species’ morphological and ecophysiological divergence. In this regard, the lowest expression levels of chloride-exporter homologues known to play a role in volume decrease after cellular swelling with kingdom-overarching conservation [46–48] were found in *C. tomentosa* SRT samples including swollen end cells (figure 1*e*). Consistent with studies by Phipps *et al*. [49], we recovered in both species homologues of a sodium transporter that were shown to be important in Charophyceaen salt resistance by their work (figure 1*f*); yet, the transporter awaits classification since it has a conserved glycine that suggests it might be a potassium transporter. While it is generally assumed that *C. baltica* is salt-sensitive [49], we would consider the possibility that both *C. baltica* and *C. tomentosa* are salt-tolerant based on the ecology of the *Chara* species investigated (both from the same brackish habitat and depth) and their genetic repertoire.

### (b) Accumulation of carotenoids in reproductive organs predated plant terrestrialization and probably originated at the base of Phragmoplastophyta

**Figure 2.**
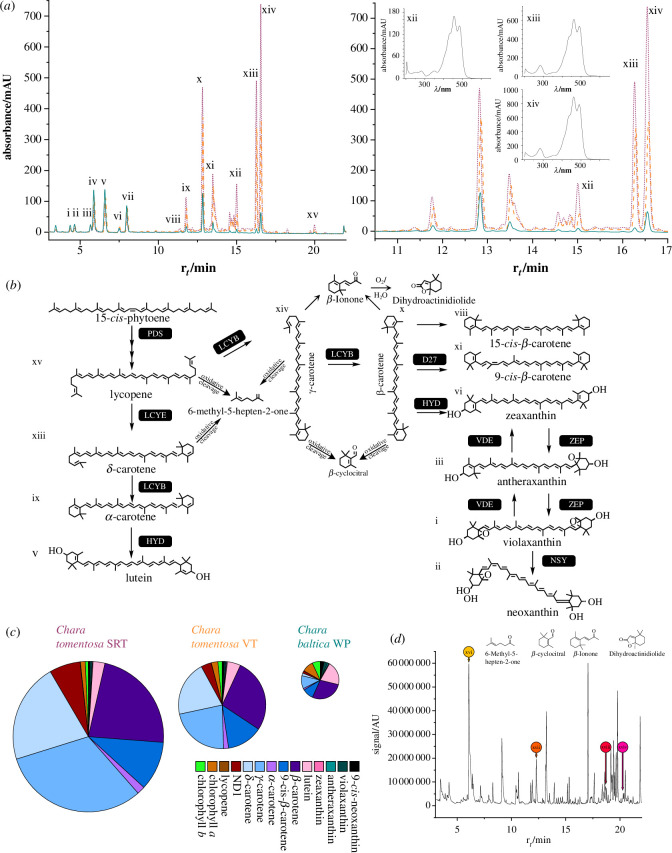
(Apo)carotenogensis in the investigated Charophyceae. (*a*) Non-normalized chromatograms of replicate 1 of the three sample groups. Purple, *C. tomentosa* SRT; orange, *C. tomentosa* VT; teal, *C. baltica* WP; i, violaxanthin ; ii, 9-cis-neoxanthin; iii, antheraxanthin; iv, chlorophyll *b*; v, lutein; vi, zeaxanthin; vii, chlorophyll *a*; viii, 15-*cis*-β-carotene; ix, α-carotene; x, β-carotene; xi, 9-*cis*-β-carotene; xii, putative torulene or some *cis*-δ-carotene or *cis*-γ-carotene; xiii, δ-carotene; xiv, γ-carotene; xv, lycopene. (*b*) Schematic of the apocarotenoid and carotenoid pathway in the investigated species. (*c*) Pigment pools of the three sample groups with area relative to pool size, legend on the left. (*d*) Headspace solid-phase microextraction coupled to gas chromatography equipped with a mass spectrometer (HS-SPME-GC-MS) chromatogram of *C. tomentosa*. Data shown were derived from VT replicate 1.

The red colour of the antheridia of many Charophyceaen algae is remarkable (see figure 1*b,c*). Schagerl & Pichler [36] found γ-carotene to be responsible for that coloration. However, the exact function of this carotenoid accumulation remained unclear. They also found γ-carotene and β-carotene to be responsible for the reddening of *C. tomentosa* VT, a mechanism especially observed in late spring/early summer [36,50]. The studies of Schagerl & Pichler [36] focused on Charophyceae from freshwater habitats. We investigated the carotenoid and chlorophyll profiles of two species of the genus *Chara* in their sexual reproductive phase, namely *C. tomentosa* and *C. baltica,* from the brackish Darss Zingster Bodden chain and compared the pigment pools with their differential gene expression profiles to further elucidate the function of carotenoid accumulation in antheridia and VT of *C. tomentosa*. Furthermore, while previous investigations of Charophyceaen carotenoid profiles used reversed phase C_18_ (RP-C_18_)-systems [36] or even normal phase separation [37], a reversed phase C_30_ high performance liquid chromatography (RP-C_30_-HPLC)-based method has the capacity to resolve structurally similar carotenoids. With the RP-C_30_-HPLC method recently published [39], we were able to detect several carotenoids in the two *Chara* species (figure 2) that were not present in Zygnematophyceae and *Physcomitrium patens* therein (cf. [39]).

**Figure 3.**
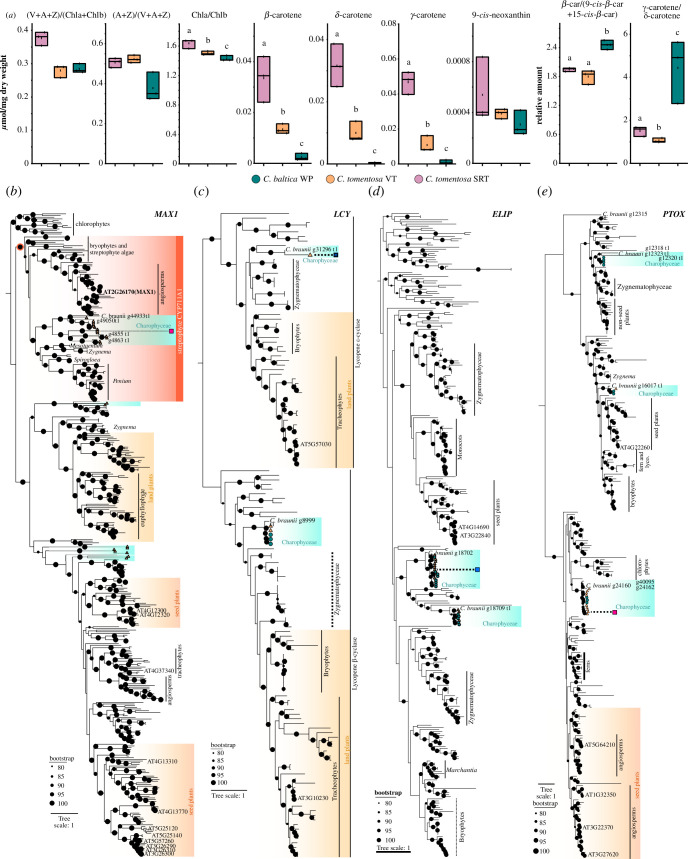
Pigment levels and ratios in *C. tomentosa* and *C. baltica*. (*a*) Individual pigment levels or pigment ratios: purple, *C. tomentosa* SRT*;* orange*, C. tomentosa* VT; teal, *C. baltica* WP. *p*-values indicate results from Kruskal–Wallis test to test for significant differences between the three sample sets. For significant results, a post hoc Conover–Iman test was performed for pairwise comparisons with p1, *C. tomentosa* SRT versus *C. tomentosa* VT; p2, *C. tomentosa* SRT versus *C. baltica*; p3, *C. tomentosa* VT versus *C. baltica* WP. (*b*–*e*) Maximum-likelihood phylogenies of genes salient to carotenoid metabolism and photoprotection. Black circles are scaled and represent the ultrafast bootstrap (UFBoot) support (see legend). Teal hues label highlight sequences from Charophyceae; *C. baltica* sequences are labelled with a dark turquoise circle, *C. tomentosa* sequences are labelled with an orange triangle. Blue boxes indicate significant downregulation, and purple boxes indicate significant upregulation of a minimum of twofold in the comparison *C. tomentosa* SRT versus *C. tomentosa* VT.

Male plants of *C. tomentosa* were dissected to separate antheridia-containing tissue SRT from VT in order to distinguish if these carotenoids accumulate in the antheridia and whether the respective transcription patterns differ. As mentioned above, *C. tomentosa* is known to change the colour of VT, especially in the upper parts starting in late spring/early summer to a more reddish appearance [50]. We investigated if this originates from the same carotenoids accumulated in the antheridia. For *C. baltica* (monoecious), we analysed the WP including antheridia and oogonia. Besides α-carotene, β-carotene and its 9-*cis*-isomer (figure 2, ix–xi), two more carotenoids (figure 2, xiii and xiv) were found in all samples, but especially accumulated in the samples of *C. tomentosa* with the highest values in antheridia-containing tissue (see figures 1–3).

The red colour of Charales antheridia thus probably originates from the following five different carotenoids: α-carotene, β-carotene, 9-*cis*-β-carotene and two chemically very similar carotenoids (xiii and xiv, similar retention times and absorption spectra; see figure 2). Based on their abundance, the main colour contribution results from β-carotene and the two chemically very similar carotenoids, which were present in *C. tomentosa* SRT at 0.0335 µmol mgDW^−1^, 0.0316 µmol mgDW^−1^ and 0.0468 µmol mgDW^−1^ as compared to the 0.0025 µmol mgDW^−1^ for α-carotene and 0.0156 µmol mgDW^−1^ for 9-*cis*-β-carotene (figure 2, xiii and xiv). This shows that carotenoid accumulation in *C. tomentosa* SRT is a trait not only limited to flowering plants [51,52] but also to other, about 700-million-year-divergent Phragmoplastophyta. Since algae do not rely on pollination for sexual reproduction, an attractive function of hyperaccumulated carotenoids as known for many seed plants [26,51,52] can be excluded.

### (c) Hyperaccumulation of δ-carotene and γ-carotene in *Chara tomentosa*

*Chara tomentosa* stands out by having an overall highly reddish thallus—both regarding its SRT and VT. Judging by similarly high values in the reddish VT of *C. tomentosa* (0.0316 to 0.0468 µmol mgDW^−1^ in SRT as compared to 0.00999 and 0.0134 µmol mgDW^−1^ in VT), the same three carotenoids are also responsible for this seasonal effect of colour change (see figures 1–3). To identify the structures of these carotenoids, we respected retention time hinging on monocyclized carotenoids because of their elution between β-carotene (figure 2, x, two rings) and lycopene (figure 2, xv, no ring). We compared the absorption spectra with literature data from Gupta *et al*. [53] and identified them as δ-carotene and γ-carotene (see figure 2, xiii and xiv).

To scrutinize our structural prediction, we applied principles of retrosynthesis investigating the accumulation of apocarotenoids (oxidative breakdown products of carotenoids) in *C. tomentosa* VT by headspace solid-phase microextraction coupled to gas chromatography equipped with a mass spectrometer (HS-SPME-GC-MS; figure 2*d*). The expectation is that the profiles reflect the structures of accumulated carotenoids. Indeed, we detected especially high levels of 6-methyl-5-hepten-2-one and high levels of β-cyclocitral (see figure 2) supporting the structures of δ-carotene and γ-carotene. This refines previous findings [36], which reported only β-carotene and γ-carotene in Charophyceaen antheridia and *C. tomentosa* VT; here, the resolving power of similar molecules by the C_30_ approach (used in this study) comes to bear, which separates molecules that can usually not be separated with RP-C_18_ systems. The strongly elevated 6-methyl-5-hepten-2-one levels (correlating with the accumulation of γ-carotene) are noteworthy. The exact functions of this apocarotenoid within photosynthetic organisms (inter- and intra-organismic) are still not known, but some interspecies/kingdom interactions have been detected including plant-insect(-plant) [54–57]. Some fishes feed on *Chara* spp. [58,59]. A correlation between volatile apocarotenoids and fishes on the other hand has not been studied to our current knowledge.

### (d) High levels of *cis*-β-carotenes in *Chara tomentosa*

All samples of *C. tomentosa* that contained high levels of β-carotene showed elevated ratios of 9-*cis*-β-carotene and 15-*cis*-β-carotene compared to *C. baltica* samples (see figures 2 and 3). Carotenoids can crystalize at high concentrations in plant cells as known from lycopene and β-carotene crystals in tomato chromoplasts (for a review of chromoplast functions, see Sadali *et al*. [60]). Simultaneous accumulation of *cis*-derivatives of the respective carotenoids could prevent this mechanism by disturbing packing owing to steric hindrance. Interestingly, in *C. tomentosa* SRT samples, the highest levels of β-carotene were detected and the lowest fluctuations in β-carotene/*cis*-β-carotenes ratios were observed pinpointing tight regulation.

The presence of 9-*cis*-neoxanthin in Charophyceae is equivocal [36,61]. Our data unequivocally detect respectable levels of 9-*cis*-neoxanthin in all samples and species investigated (see figure 3). The question why 9-*cis*-neoxanthin biosynthesis genes are not detectable in *Chara braunii* [61] remains to be solved in future studies.

### (e) Carotenoid-derived metabolites and volatile apocarotenoids in Charophyceae

**Figure 4.**
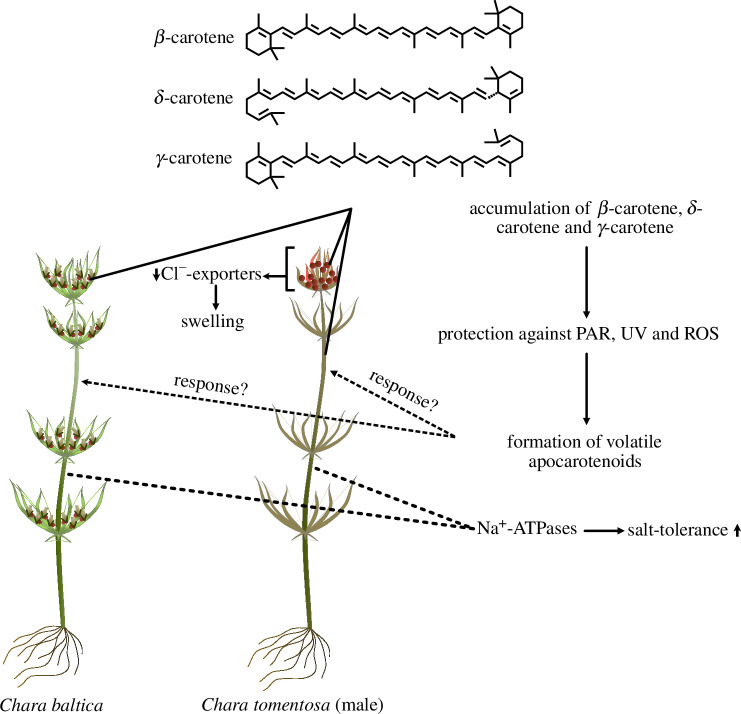
Ecophysiological model for *C. baltica* and *C. tomentosa* (male). Small arrows indicate elevation or depletion.

Apocarotenoids have diverse biological functions in plants, ranging from stress signalling to interspecies communication [26,62,63]. We detected the volatile apocarotenoids β-ionone and dihydroactinidiolide (figures 2 and 4). They probably completely originate from non-enzymatic cleavage since no CAROTENOID CLEAVAGE DIOXYGENASE (CCD; main enzymes of oxidative carotenoid cleavage) homologous sequences were found in the genome of *C. braunii* and also not in our transcriptomes. Several other carotenoids of unknown identity were detected, eluting in between β-carotene and δ-carotene (see figure 2). One of them showed slightly higher levels, comparable to α-carotene especially in *C. tomentosa,* and was included as ND1 (figure 2, xii) in the overall pool. The absorption spectrum was similar to δ-carotene and γ-carotene with a shift to a shorter wavelength; the absorbance spectrum included an additional peak. Based on this, the structure is most likely similar to torulene (assumed for quantification) or a *cis*-δ-carotene or *cis*-γ-carotene but needs further structural investigations.

To understand the molecular chassis for—and acting on—the large pool of carotenoids in the SRT of *C. tomentosa,* which also contributes to apocarotenoid abundance, we screened for homologues of genes that code for proteins involved in carotenoid biosynthesis and metabolism. Most homologues were not significantly different in their transcript levels and, if so, they were depleted (electronic supplementary material, table S1). The gene with the most strongly elevated expression in SRT versus VT was a homologue of a cytochrome P450, CYP711A1 (*C. tomentosa* TRINITY_DN2319_c1_g1_i17; 23.25-fold higher, Benjamini–Hochberg corrected *p*-value of 0.0236). CYP711A1 is also known as MORE AXILLARY BRANCHES 1 (MAX1) and is a key enzyme in strigolactone biosynthesis that converts cleavage products of carotenoids into carlactonic acid [64]. We computed a phylogeny of all homologues of MAX1 that we detected in *C. tomentosa* and additionally sampled homologous sequences from across the green lineage (figure 3*b*). The sequences from *C. tomentosa* are clear co-orthologues of MAX1 (figure 3*b*). Indeed, strigolactones have been reported from Charophyceae [65]—but their presence has remained enigmatic. Yet the situation is more complicated, as MAX1-independent production of strigolactone(-like) metabolites occurs in the moss *P. patens* [66]—which completely lacks MAX1, a lack that we also recovered in our phylogeny (figure 3*b*). At the moment it appears that strigolactones have been recruited multiple times, but probably in embryophytes as ancient signalling molecules in symbiosis [67]. We interpret the MAX1 (co-)orthologues and the enriched expression of some of these in *C. tomentosa* SRT as a hint towards diverse carotenoid-derived molecules arising where carotenoids abound as substrates—be it strigolactone(-like) molecules or different ones. The apocarotenoids detected here are a point in case, but probably there is a diverse bouquet of carotenoid-derived metabolites emerging in Charophyceae—including by the aid of enzymes such as CYP711A1-likes.

An enrichment of certain carotenoids can not only be explained by the high expression of enzymes that yield products but also by the low expression of enzymes that use them as substrates. The altered levels of monocyclized and double-cyclized carotenoids were reflected in LCY expression levels. We computed a phylogeny of LCYs and recovered clear clades of LCYE (epsilon-ring formation) and LCYB (beta-ring formation) homologues (figure 3*c*). Here, the LCYE homologue of *C. tomentosa* stood out as its expression levels were depleted (in some cases almost to 0) in antheridia-rich *C. tomentosa* SRT, compared to VT (figure 3*c*). This could explain the accumulation of these ‘intermediate’ carotenoids in α-carotene and β-carotene biosynthesis. It furthermore illustrates how during evolution the same enzyme chassis can yield starkly different metabolite phenotypes.

### (f) Ecological implications of the pigment pools of *Chara tomentosa* and *Chara baltica*

The overall size of the pigment pools of investigated Charales species and tissues (figure 2) reflects their appearance. *Chara tomentosa* changes the colour of its upper thalli to a more reddish appearance starting in late spring/early summer, while *C. baltica* grows in about the same depth but does not alter its colour. On carotenoid and chlorophyll levels, this is reflected by the larger overall pigment pool of *C. tomentosa* with the largest pool in samples containing SRT related to carotenoid hyperaccumulation detected in antheridia (see figure 2). A higher accumulation of carotenoids with absorbance shifted to longer wavelengths, namely δ-carotene and γ-carotene, was observed in *C. tomentosa* (see figures 2 and 3). The seasonal change of the colour of *C. tomentosa* VT originating from carotenoid accumulation aligns with increased photosynthetically available radiation (PAR) and longer photoperiods starting in late spring/early summer. This mechanism is probably beneficial for withstanding the characteristics of seasonal change. *Chara tomentosa* is found in brackish as well as freshwater habitats, while *C. baltica* is found only in brackish habitats. Freshwater habitats often undergo fluctuations in water depth sometimes correlated to season. As water depth increases, overall light intensity decreases, and additionally, the composition of PAR changes with the most rapid decrease in intensity of red light, followed by orange, green and blue [68].

The hyperaccumulated carotenoids detected in *C. tomentosa* might also help survival in differing habitats. Accumulation alone does not distinguish between a photoprotective and a light-harvesting function of carotenoids (for a review of carotenoid functions, see Nisar *et al*. [12]). The xanthophyll pool (Violaxanthin + Antheraxanthin + Zeaxanthin [V + A + Z]/Chlorophyll *a* + Chlorophyll *b* [Chl*a* + Chl*b*], *p*-value = 0.066) and the de-epoxidation state (A + Z/V + A+Z, *p*-value = 0.061) show no significant difference between the groups. This hints at a similar amount of photo-oxidative stress in the organisms and taking the absolute range into account a small overall amount of photo-oxidative stress. Meta-analyses have shown that these values are good indicators for observed photo-oxidative stress and V + A + Z/Chla + Chlb can be seen as a light ‘memory’ [69]. Since mature antheridia are not photosynthetically active, a photoprotective function of accumulated carotenoids is way more likely. Xanthophyll pigments are usually used for light harvesting in PSII, not carotenes (for a review of carotenoid functions also see Esteban *et al*. [70]). This leads to the assumption that also in VT of *C. tomentosa,* the hyperaccumulation probably has a photoprotective function. Furthermore, carotenoids xii, xiii and xiv (figure 2) show small but significant absorbance in the range of UVB radiation (and xii even additionally in the range of UVA; see figure 2) and *cis*-β-carotenes in the range of UVA. Owing to the sheer immense amount of these carotenoids in antheridia of *C. tomentosa* SRT and *C. tomentosa* VT, a UV protective function is likely. This is consistent with the seasonal increase in UV radiation. Given the detrimental effect of UV and strong light on DNA, it is conceivable that the accumulation of UV-absorbing carotenoids serves as a protection mechanism of the germline in the SRT. This accumulation of carotenoids (and other antioxidants) as protectants of DNA against UV and strong light in SRT is a widely shared feature of many seed plants including high levels of lycopene in tomatoes [71] and of carotenes and lutein and zeaxanthin in maize kernels [72] to just name some examples. The question remains if this trait evolved in the last common ancestor (LCA) of all Phragmoplastophyta and was lost in some lineages (e.g. Zygnematophyceae [73]) or evolved independently in the LCA of Charophyceae and the LCA of land plants (or even specific land plant lineages).

*Chara baltica* does not accumulate carotenoids to the same extent as *C. tomentosa*, given that its pool is smaller than both *C. tomentosa* SRT and VT (figure 2*c*). To dissect the possible molecular consequences of this large pool in *C. tomentosa*, we examined differential gene expression of light stress-related genes. Consistent with a putative photoprotective function of the accumulated carotenoids, the expression of early light-inducible proteins (ELIPs) was significantly lower expressed in SRT versus VT of *C. tomentosa* (figure 3*d*). ELIPs are among the classical land plant light-stress-induced protective proteins by their chlorophyll *a*/*b* and LHC binding properties [74,75]. Those were also shown to be important stress responses to diverse conditions in other Phragmoplastophyta, namely Zygnematophyceae [11,39,76]. A recently emerging player in stress response, especially in high-light stress situations, and simultaneously essential for carotenogenesis are so-called plant terminal oxidases (PTOXs) [72,77]. We recovered homologues of genes probably coding for these essential enzymes; their expression was significantly enriched in SRT versus VT (figure 3*e*), correlating with the observed carotenoid pool sizes. PTOXs essential role in regulating the carotenoid flux and accumulation has also been shown for maize kernels [72]. The conservation of this regulatory function can be rationalized by the need for the electron-accepting cofactor plastoquinone for the multiple desaturation steps of the carotenoid pathway (before cyclization), which is re-oxidized by PTOXs [78,79]. It is noteworthy that our phylogenetic analyses show that Charophyceae harbour a diversified set of PTOXs, which includes homologues falling into a clade of sequence homologues only found in non-seed plants and streptophyte algae (figure 3*e*).

## 3. Conclusion

Sexual reproduction is intertwined with oxidative homeostasis. ROS are essential signalling molecules [80] in the development and function of reproductive organs [81,82]. ROS can also be culprits, e.g. emerging from photosynthesis and causing oxidative damage to the photosystem complexes [83,84]. Similarly, the reproductive organs of land plants, foremost flowers of angiosperms, are often highly pigmented, and the function of this pigmentation includes, next to pollinator attraction, also (photo-)protection [85,86]. Indeed, transcriptome analysis of the reproductive structures of *C. braunii* showed differential regulation in genes salient to ROS homeostasis, especially peroxidases [61].

Here, we studied two sympatric species of *Chara*, *C. baltica* and *C. tomentosa* (figure 4). We recovered genetic signatures of the ecophysiology of the two species, for example, swelling control by chloride exporters and enriched PTOX in *C. tomentosa*. We found β-carotene, δ-carotene and γ-carotene to accumulate in *Chara* antheridia and in *C. tomentosa* VT besides several other carotenoids of unknown identity. These carotenoids should be addressed in future studies. Our data suggest that the accumulated carotenoids contribute to photoprotection. However, exact molecular functions and associated mechanisms need to be further investigated. From this carotenoid pool, volatile apocarotenoids emerge, which were not reported previously in Charophyceae—with some in extensive levels hinting at a putative function in stress response or dormancy—subjects that deserve future investigation.

Overall, our work provides an example of how from a conserved chassis of enzymes and metabolites, a unique phenotype can arise: hyperaccumulation of conserved, but usually fast-metabolized carotenoids. This increased pool of carotenoids probably yields a divergent bouquet of carotenoid-derived metabolites (including apocarotenoids) that await exploration. Conceivably, this hyperaccumulation phenotype arose during evolution through minimal changes in expression patterns. At the same time, the accumulation of carotenoids in reproductive structures is a trait that is found in many streptophytes, suggesting that there is a biological programme that is shared across more than 700 million years of phragmoplastophyte evolution.

## 4. Material and methods

### (a) Algal species

*Chara tomentosa* L. 1753, a dioecious and diplostichous green–greyish charophyte occurs, in brackish and freshwater (figure 1). Characteristic features of this species are the reddening of the upper thallus part during its life cycle and the swollen end cells at the branchlets [37]. The sites are usually dominated by male plants. Their antheridia can reach a diameter of up to 1425 µm [37]. *Chara baltica* (Hartman) Bruz. 1824 is a monoecious diplostichous to triplostichous charophyte of light to dark green colour (figure 1). This species can only be found in brackish habitats. In contrast to *C. tomentosa*, where gametangia are usually only formed on the uppermost segments, *C. baltica* develops gametangia on all segments (figure 1). Antheridia are 2–3 times smaller than those of *C. tomentosa* with a size of 500 to 700 µm [37].

### (b) Sampling

Plant material of *C. tomentosa* and *C. baltica* from Michaelsdorf (54.371306° N, 12.569778° E), a lagoon in the Bodstedter Bodden of the Darss-Zingst Bodden chain, was used for this study. This site is characterized by a high proportion of organic suspended matter, which strongly affects the light climate and underwater vegetation. The sheltered part of the lagoon has a soft muddy bottom of up to 1 m and is covered with charophytes of *C. tomentosa*, *C. baltica*, *Chara aspera* and *C. baltica* var. *liljebladii*.

The plants of both species were collected by hand in the year 2023 at a depth of 50–75 cm. Male plants of *C. tomentosa* had developed antheridia. Harvested plants of *C. baltica* exhibit antheridia and oogonia but no oospores. For all following analyses, VT or top parts (antheridia + swollen end cells at the top of the plant) for *C. tomentosa* were used, and WP samples (VT + antheridia and oogonia) for *C. baltica* were used.

Physico-chemical parameters were measured during sampling as follows: temperature 16°C, salinity 4.2 ‰, conductivity 11520 μs cm^−1^ (measured with a commercial device for home aquaculture). Plants were transported in their native water medium.

### (c) Chemicals

A detailed description of the analytical standards used for peak identification and calibration of the analytical instruments can be found in Rieseberg *et al*. [39]. The solvents used for the extraction of pigments and for performing the HPLC analysis are also described therein.

### (d) Carotenoid and chlorophyll extraction

A detailed description of the extraction of carotenoids and pigments is published in Rieseberg *et al*. [39]. In short, *ca* 10 mg of lyophilized tissue stored at −80°C under an argon atmosphere prior to extraction was refrozen in liquid N_2_ and afterwards extracted with 700 μl acetone : water (80 : 20 + 0.1 wt% butylated hydroxytoluene [BHT]) and with 700 μl acetone (+ 0.1 wt% BHT) at 4°C room temperature with dimmed lights. Samples were analysed immediately after extraction.

### (e) High-Performance Liquid Chromatography with Ultraviolet-Visible detection via a Diode Array Detector (HPLC–UV–Vis–DAD) measurements of pigments

The method for pigment analysis was previously described in Rieseberg *et al*. [39]. In short, the HPLC system Agilent 1100 series with a Ultraviolet-Visible detection via a Diode Array Detector (UV–Vis–DAD) equipped with a YMC Carotenoid C30 column (250 × 4.6 mm inner diameter [ID] S-3 µm) from YMC Europe (20°C). Eluents of the 26 min gradient were eluent A (methanol : water, 98 : 2), eluent B (methanol : water, 95 : 2) and eluent C (Methyl *tert*-butyl ether). For the exact gradient and the description of the quantification, see in general Rieseberg *et al*. [39]. ND1 (putative torulene), δ-carotene and γ-carotene were quantified at 451 nm based on β-carotene calibration since no commercial standards were at hand to include specific calibration. Identification of δ-carotene and γ-carotene was based on comparisons with absorption spectra published in Gupta *et al*. [53] (also see the electronic supplementary material and figure 2). Additionally, retention times and principles of retrosynthesis from detected apocarotenoids were taken into consideration.

Statistical analyses of metabolite data were performed with OriginPro 2020. If Kruskal–Wallis–ANOVA showed a significant difference among the groups (*p*‐value < 0.05), a post hoc Conover–Iman’s test was performed for individual comparisons.

### (f) Headspace solid-phase microextraction coupled to gas chromatography equipped with a mass spectrometer measurements of apocarotenoids

The method described [39] was operated in scan mode (*m*/*z*: 40–300) to identify volatile apocarotenoids of *C. tomentosa*. Apocarotenoids were identified by comparison of retention times and fragmentation patterns of the commercial standards with in planta fragmentation patterns. Frozen and lyophilized tissue (20–40 mg) of *C. tomentosa* VT was homogenized with a sharp spatula and transferred into a brown-glass headspace vial and the analysis protocol of the GC-MS started. For details of the temperature gradient, technical compartments, etc. and the fragmentation patterns of commercial standards of the apocarotenoids from figure 2, see Rieseberg *et al*. [39].

### (g) RNA extraction

RNA extraction was based on Dadras *et al*. [76] and only slightly modified. Frozen tissue of *Charas* was stored at –80°C prior to extraction, was refrozen in liquid N_2_, homogenized with a spatula and afterwards mixed with 1 ml of lysis buffer containing 2-mercaptoethanol (10 μl ml^−1^), vortexed and transferred for 4 min to an ultrasonic bath. Next, heat shock (56°C) for 5 min was applied. Protocol B (increased binding solution (750 μl)) of the Sigma-Aldrich Spectrum Plant Total RNA Kit was carried out as described by the vendor.

### (h) RNAseq and transcriptome assembly

At Novogene (Cambridge, UK), the samples underwent quality checks using a Bioanalyzer (Agilent Technologies Inc., Santa Clara, CA, USA), and library preparation was performed based on polyA enrichment and using directional mRNA library preparation. The libraries were quality checked and sequenced using the NovaSeq 6000 platform (Illumina) with Novogene dual adapters: 5′- AGATCGGAAGAGCGTCGTGTAGGGAAAGAGTGTAGATCTCGGTGGTCGCCGTATCATT-3′ for read 1 and 5′- GATCGGAAGAGCACACGTCTGAACTCCAGTCACGGATGACTATCTCGTATGCCGTCTTCTGCTTG-3′.

Read quality was addressed by using FastQC [87]. For this study, four different de novo assemblies were conducted using Trinity v.2.15.1 [43] after adapter trimming with Trimmomatic [88] (--trimmomatic ‘ILLUMINACLIP:novogene_adapter_sequences.fa:2:30:10:2:keepBothReads LEADING:3 TRAILING:3 MINLEN:36’). The four sets consist of the *C. tomentosa* sets: ‘SRT’, ‘VT’ and a combination of these two sets ‘TOR’. Another set was created based on the WP transcriptome of *C. baltica* called ‘BCA’. The completeness of the transcriptomes was assessed with BUSCO v5.4.3 [89] using the ‘eukaryota_odb10’ reference set. The BUSCO completeness of all newly assembled transcriptomes was on average 89.53%. Protein-coding genes were identified using Transdecoder v5.5.0 [90], using the programme’s defaults. For the differential expression analyses, we used the ‘TOR’ set assemblies and protein file.

### (i) Decontamination

Because the samples were not from an axenic origin, we wanted to remove potential decontaminants. We conducted this by doing sequence similarity searches against a comprehensive database that included proteins from various sources. These sources include the *C. braunii* S276 genome [61] as well as potential contaminants such as RefSeq [91] representative bacterial genomes (210 042 521 proteins), fungi (4 990 228 proteins), viruses (644 246), invertebrate (8 686 952), mitochondrion (240 117), plasmid (2 080 798), plastid (1 064 130) and protozoa (1 164 439). We employed MMseqs2 [92] for the search, using an iterative approach with increasing sensitivities and maintaining a maximum of 10 hits (--start-sens 1 --sens-steps 3 -s 7 --alignment-mode 3 --max-seqs 10). To ensure stringent decontamination, we retained, with the help of Get Positive DataSet (GPDS) [93].

### (j) Differential expression analyses

To understand the profile of the three different sets (*C. tomentosa* SRT, *C. tomentosa* VT, and *C. baltica*), a PCA was performed according to the methods described by Dadras *et al*. [76]. We followed this up by doing a quantification of the transcript and gene abundance. We started by running the Trinity ‘align_and_estimate_abundance.pl’ and using Kallisto [44] as its alignment-free quantification method. Abundances were estimated with the Trinity script ‘abundance_estimates_to_matrix.pl’. Additionally, we wanted to know how many number of expressed transcripts or genes there were, per TPM value. That is why we followed up with the two additional Trinity scripts. ‘count_matrix_features_given_MIN_TPM_threshold.pl’ and ‘count_matrix_features_given_MIN_TPM_threshold.pl’.

This resulted in 989 207 possible transcripts for *C. tomentosa* and 1 367 824 for *C. baltica*. Since this was an unlikely high number for meaningful transcripts in a transcriptome, we conducted several filtering steps based on the minimum expression levels of 1, 3 and 8 TPM with the Trinity script ‘filter_low_expr_transcripts.pl’ followed by ‘get_Trinity_gene_to_trans_map.pl’. For these three new fasta files proteins were identified with Transdecoder and decontaminated, this was followed up by transcript quantification as previously described. Differential expression analyses were done with the Trinity script ‘run_DE_analysis.pl’ and the differential expression analyses method of DESeq2 [45]. Additionally, we did a transcriptome functional annotation analysis with Trinotate [94]. For this, we conducted a BLASTp and blastx against the UniProtKB/Swiss-Prot database [95], Infernal [96], HHMer [97] with the Pfam Database [98] and eggnog-mapper V2 [99]. The results were loaded into a Trinotate database, and a Trinotate report was generated. We then used DEMC (de novo tool) to extract several files (several trinotate-derived Kyoto Encyclopedia of Genes and Genomes (KEGG)-related files [pathway, module, KEGG Orthology {KO}], differential expressed transcripts and proteins in fasta format and a differential expressed relevant Trinotate output). With the help of KEGGCounter (de novo tool), we filtered out irrelevant pathways and created a KEGG matrix file.

### (k) Phylogenetic analysis

To understand the evolutionary history of several genes of interest, we used: (i) the homologues we detected in the predicted proteomes of the two *Chara* species investigated here (*C. tomentosa* and *C. baltica*), and (ii) well-characterized Arabidopsis proteins as a query in a BLASTp search against a protein database inferred from the genomes of: *Arabidopsis thaliana* (Lamesch *et al*. [100]), *Arabidopsis lyrata* (V2.1; [101,102]), *Brassica oleracea* [103], *Brassica rapa* (FPsc v1.3; [104]), *Capsella grandiflora* [105], *Triticum aestivum* [106], *Carica papaya* [107], *Oryza sativa* (v7.0; [108]), *Brachipodium distachyon* (v3.2; [109]), *Amborella trichopoda* (v1.0; [110]), *Gossypium hirsutum* [111], *Theobroma cacao* [112], *Solanum lycopersicum* [113], *Nicotiana tabacum [114]*, *Gnetum montanum* [115], *Picea abies* [116], *Azolla filiculoides* [117], *Isoetes taiwaniensis* [118], *Selanigella moellendorffi* [119], *P. patens* [120], *Sphagnum fallax* [121], *Marchantia polymorphya* [122], *Anthoceros punctatus* [123], *Anthoceros agrestis* [123], *Mesotaenium endlicherianum* (v1 [124] and v2 [76]), *Penium margaritaceum* [125], *Spirogloea muscicola* [124], *C. braunii* [61], *Klebsormidium nitens* (v1.1; [126]), *Chlorokybus melkonianii* ([127]—for naming, see Irisarri *et al*. [128]), *M. viride* [127], *Micromonas pusilla* (v3.0; [129]), *Ostreococcus lucimarinus* (v2.0; [130]), *Ulva mutabilis* [131], *Chlamydomonas reinhardtii* (v5.6; [132]) and *Chlorella variabilis* (Blanc *et al*. [133])—as well as against the two proteomes predicted for *C. tomentosa* and *C. baltica*. All significant hits (*e*-value cut-off of 10^−5^; in the case of larger gene families, we applied a bit score cut-off of 100) were aligned with MAFFT v7.490 [134] (L-INS-I). We computed maximum-likelihood trees using IQ-Tree v.1.5.5 [135] with 1000 ultrafast bootstrap pseudo-replicates [136]. The best models for protein evolution were determined by ModelFinder [137], and the best model according to the Bayesian information criterion was used.

## Data Availability

All RNA sequencing data have been uploaded to NCBI SRA under Bioproject PRJNA1088485. The predicted proteins and data on the phylogenetic analyses are available on Zenodo [138]. The computational analyses are outlined under [139]. Supplementary material is available online [140].
